# A rare case of symmetrical drug‐related intertriginous and flexural erythema with tamoxifen use

**DOI:** 10.1002/ccr3.9401

**Published:** 2024-08-28

**Authors:** Syed Ather Hussain, Ryan Berenji, Sarah Faisal, Liaqat Ali, Ashley Knavel

**Affiliations:** ^1^ Department of Medical Oncology Roswell Park Comprehensive Cancer Center Buffalo New York USA; ^2^ General Physician Cancer Care Williamsville New York USA; ^3^ Jinnah Sindh Medical University Karachi Pakistan; ^4^ Upstate Dermatopathology Williamsville New York USA; ^5^ Department of Dermatology Buffalo Medical Group Williamsville New York USA

**Keywords:** baboon syndrome, SDRIFE, tamoxifen, type IV hypersensitivity

## Abstract

Symmetrical drug‐related intertriginous and flexural erythema is characterized by a diffuse symmetric rash of the gluteal and intertriginous areas with only one published report of association with tamoxifen. It is imperative for clinicians to recognize tamoxifen‐induced SDRIFE to avoid life‐threatening dermatologic complications, which can be resolved with discontinuation of tamoxifen.

## 1 INTRODUCTION

Symmetrical drug‐related intertriginous and flexural erythema (SDRIFE) is an infrequent type IV hypersensitivity reaction, characterized by a diffuse and symmetric erythematous maculopapular rash of the gluteal areas, along with involvement of one other intertriginous area, in the absence of systemic symptoms.^1^ The classical histopathology shows superficial perivascular infiltrates with spongiosis.^2^ It has been associated with certain antibiotics, anti‐hypertensives, and contrast agents.^3^

Tamoxifen is a selective estrogen receptor modulator that blocks estrogen receptor in the breast.^3^ Side effects related to tamoxifen use include hot flashes, venous thromboembolism and uterine cancer. There has only been one other published report of tamoxifen‐induced SDRIFE.^3^

## 2 CASE PRESENTATION

A 48‐years‐old premenopausal female with no past medical history including no food allergies, was diagnosed with right breast carcinoma after an abnormal screening mammogram and biopsy. She underwent a lumpectomy with sentinel node biopsy, which showed an invasive 8 mm tumor, negative margins and no nodal involvement, with positive estrogen (95%) and progesterone receptor (95%) but negative human epidermal growth factor receptor 2 (HER2) (1+). Her Oncotype DX score was low, so adjuvant chemotherapy was not recommended. She was initiated on tamoxifen 20 mg daily after completing adjuvant radiation.

**FIGURE 1:**
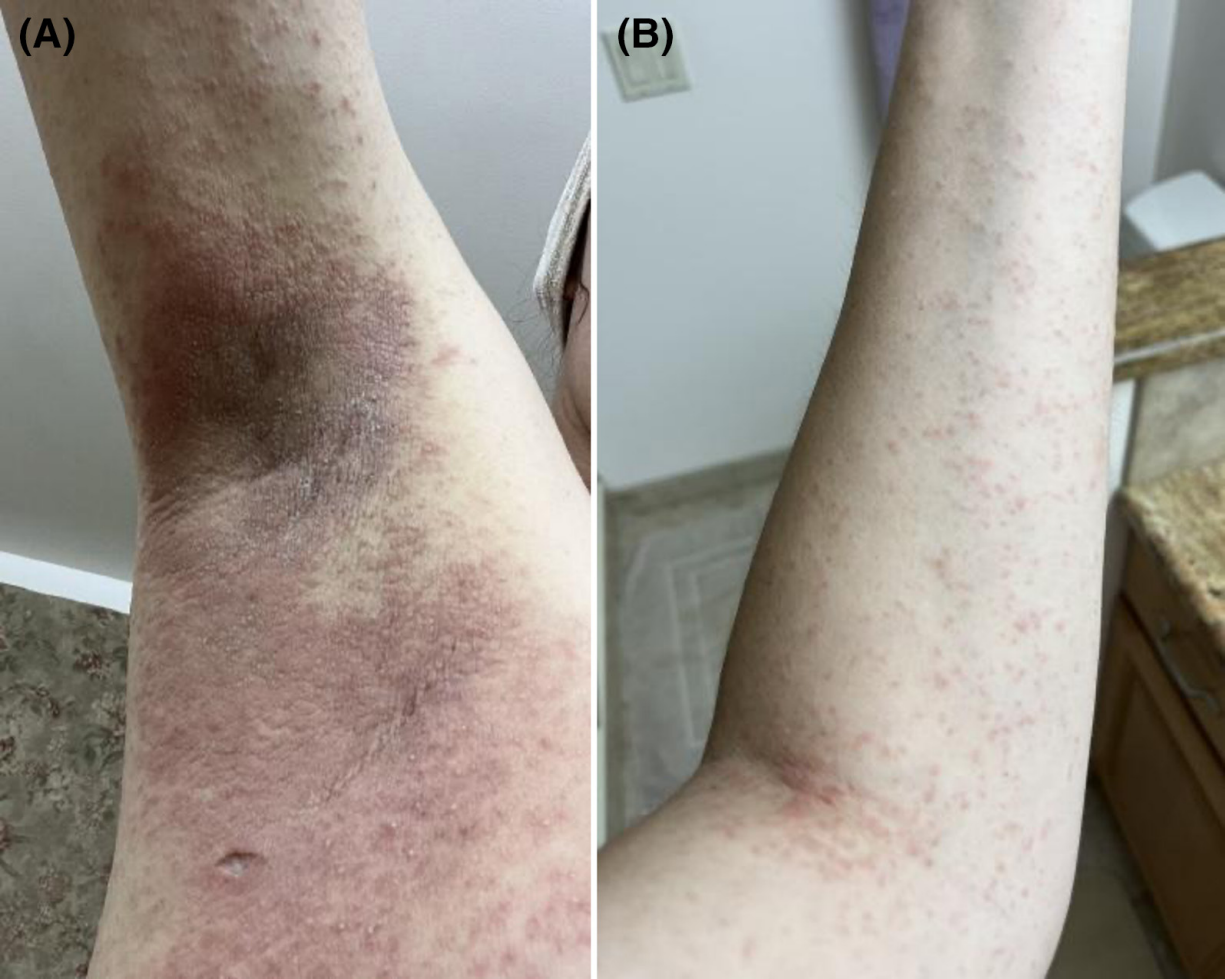
Papulovesicular rash due to tamoxifen involving bilateral axilla (A) and forearm antecubital fossa (B).

**FIGURE 2:**
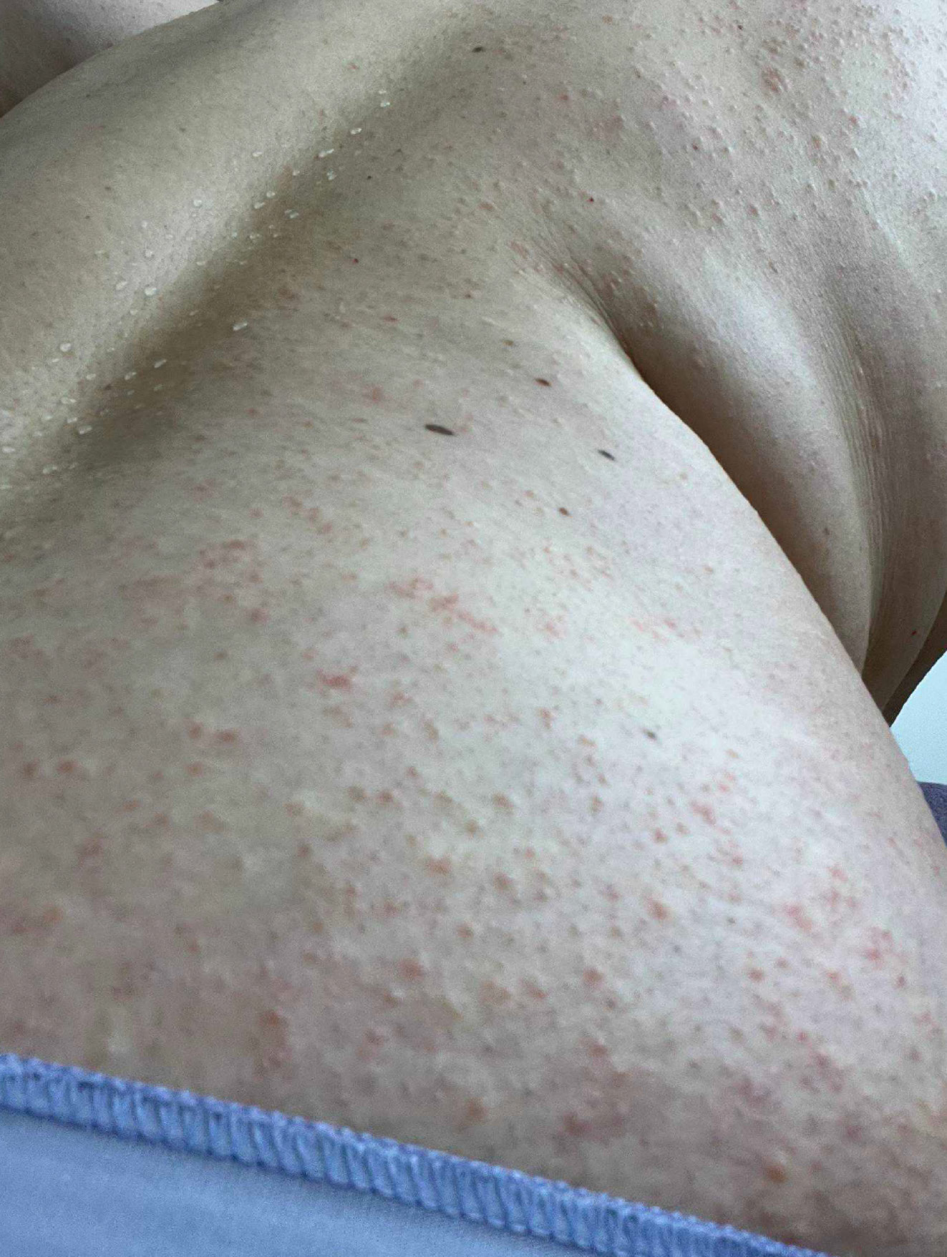
Papulovesicular rash on the lower back and the buttock.

**FIGURE 3:**
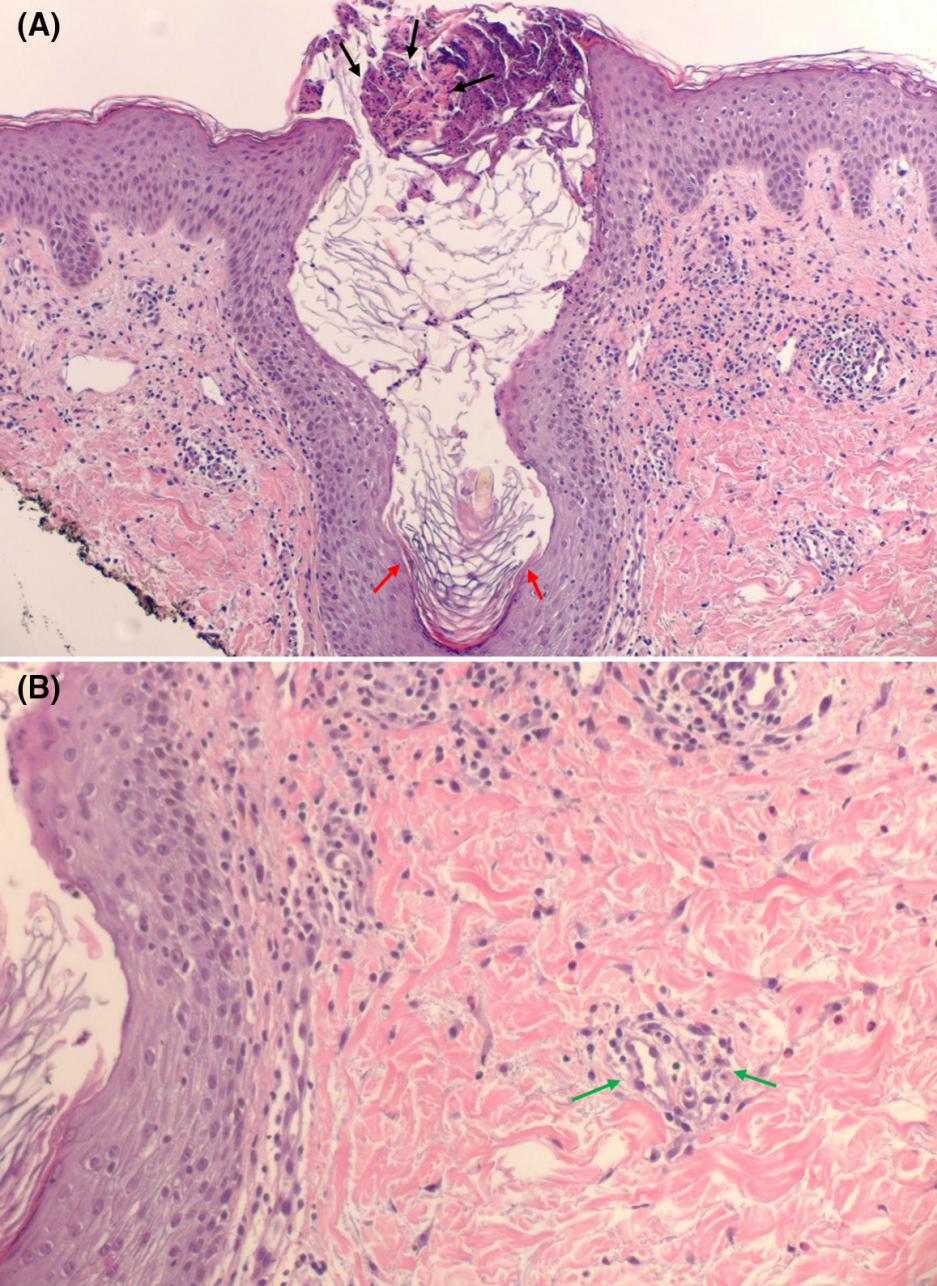
Skin biopsy demonstrates eosinophilic and lymphocytic exocytosis, epidermal spongiosis, neutrophils forming pustules within stratum corneum (black arrows) and dilated hair follicles (red arrows) (A) accompanied by papillary dermal edema and eosinophil rich inflammatory infiltrates around the vessels (green arrows) (B). (H & E, ×200).

After taking tamoxifen for 9 months, she developed a pruritic erythematous papulovesicular rash in the axilla bilaterally (Figure 1A). She initially tried topical steroids and oral antihistamines with no improvement. The rash slowly spread to her neck, chest, abdomen, extremities, buttocks, and groins symmetrically, covering more than 90% of her body (Figures 1B and 2). Her skin biopsy revealed significant epidermal spongiosis with vesicle formation, papillary dermal edema and perivascular eosinophil rich infiltrates with neutrophils forming pustules within stratum corneum and dilated hair follicles (Figure 3A,B).

Given the symmetric papulovesicular eruption, absence of systemic manifestations including oral or mucosal involvement, and biopsy results, her rash was suggestive of SDRIFE associated with tamoxifen use.

She stopped tamoxifen and received intramuscular methylprednisolone 40 mg once followed by a tapering dose of oral prednisone 40 mg daily, along with topical steroids. After 10 days of stopping tamoxifen, her debilitating pruritic rash completely resolved. Adjuvant endocrine therapy was switched to goserelin and letrozole to decrease her risk of breast cancer recurrence.

## 3 DISCUSSION

In the previously published case of tamoxifen‐induced SDRIFE, the patient had a symmetric papulovesicular rash on the face, arms, legs, back, chest, axilla, buttocks, and inguinal areas. The rash had appeared several months after initiation of tamoxifen like our case, suggestive of a delayed hypersensitivity reaction and resolved quickly after discontinuation of tamoxifen.^3^ Skin biopsy revealed focal hydropic degeneration of dermo‐epidermal junction, lymphocytic exocytosis, and dermal perivascular lymphocytic infiltration and edema.

The differential diagnoses for SDRIFE include acute generalized exanthematous pustulosis (AGEP) and drug rash with eosinophilia (DRESS), which present with systemic symptoms, unlike SDRIFE.^3^ Given the widespread use of tamoxifen in the treatment of breast cancer, it is imperative for clinicians to promptly recognize tamoxifen‐induced SDRIFE, to avoid potentially life‐threatening dermatologic complications, which can easily be resolved with discontinuation of tamoxifen.

## AUTHOR CONTRIBUTIONS

**Syed Ather Hussain**: Conceptualization; writing—original draft. **Ryan Berenji**: Writing—original draft. **Sarah Faisal**: Writing—original draft. All authors have approved the submitted manuscript.

## FUNDING INFORMATION

The authors received no specific funding for this work.

## CONFLICT OF INTEREST STATEMENT

The authors declared no potential conflicts of interest with respect to the research, authorship, and/or publication of this manuscript.

## ETHICS STATEMENT

This report for a clinical image was conducted in accordance with the Declaration of Helsinki. The collection and evaluation of all protected patient health information was per‐formed in a Health Insurance Portability and Accountability (HIPAA) complaint manner.

## CONSENT

Written informed consent was obtained from the patient to publish this report in accordance with the journal's patient consent policy.

## Data Availability

Data sharing not applicable to this article as no data sets were generated or analyzed during the current study.
